# Simulating Lightning‐Induced Tree Mortality in the Dynamic Global Vegetation Model LPJ‐GUESS

**DOI:** 10.1111/gcb.70312

**Published:** 2025-06-24

**Authors:** Andreas Krause, Konstantin Gregor, Benjamin F. Meyer, Anja Rammig

**Affiliations:** ^1^ Technical University of Munich TUM School of Life Sciences Freising Germany

**Keywords:** atmosphere‐vegetation interactions, carbon cycle, disturbance, ecosystem modelling, flash, forest dynamics, lightning strike, tree death

## Abstract

Lightning is an important yet often overlooked disturbance agent in forest ecosystems. Recent research conducted in Panama suggests that lightning is a major cause of large tree mortality in tropical forests. However, lightning‐induced tree mortality is not included in state‐of‐the‐art ecosystem models. Here, we implement a general lightning mortality module in the dynamic global vegetation model LPJ‐GUESS to explore the impacts of lightning on forests at local and global scales. Lightning mortality was implemented stochastically in dependency of local cloud‐to‐ground lightning density and simulated forest structure based on findings from the Panamanian forest. For this site, LPJ‐GUESS adequately simulates the average number of trees of different size classes killed per lightning strike, with a total of 2.9 simulated versus 3.2 observed. The model also captures the estimated contribution of lightning to the overall mortality of large trees (21% simulated vs. 24% observed). Applying the new model version to other tropical and temperate forests for which observation‐based estimates on lightning mortality exist, LPJ‐GUESS reproduces estimated impacts in some forests but simulates substantially lower impacts for others. Global simulations driven by two alternative products of cloud‐to‐ground lightning densities suggest that lightning kills 301–340 million trees annually, thereby causing 0.21–0.30 GtC yr.^−1^ of dead biomass (2.1%–2.9% of total killed biomass). The simulations also reveal that the global biomass would be 1.3%–1.7% higher in a world without lightning. Spatially, simulated lightning mortality is largest in the tropical forests of Africa. Although our simulations suggest an important role of lightning in forest ecosystems on a global scale, more data on lightning‐induced tree mortality across different forest types would be desirable for more accurate model calibration and evaluation. Given the anticipated increase in future lightning activity, incorporating lightning mortality into ecosystem models is needed to obtain more reliable projections of terrestrial vegetation dynamics and carbon cycling.

## Graphical abstract

Lightning plays a bigger role in killing trees than many people realize, especially in the tropics. We have added lightning‐induced tree mortality to a global vegetation model, showing it could kill over 300 million trees annually and significantly affect forest structure and carbon storage. The results highlight the need to include lightning mortality in forest and climate models, especially as lightning activity is expected to increase with climate change.

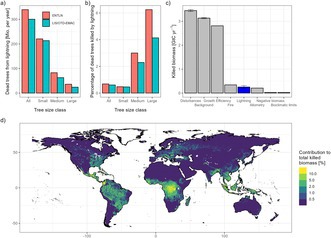

## 1 Introduction

Forest ecosystems currently store 870 ± 61 GtC in vegetation and soils and take up an additional 3.5 ± 0.4 GtC each year, equivalent to 45% of anthropogenic fossil fuel emissions (Pan et al. 2024). The balance between carbon assimilation (i.e., photosynthesis) and carbon loss (i.e., mortality and turnover) determines how much carbon is sequestered in biomass. Understanding the drivers of tree mortality is thus crucial to adequately project forest dynamics and carbon storage in a changing climate. However, there is still a considerable knowledge gap of how and why trees die (Senf et al. 2025). Pugh et al. (2019) estimated that stand‐replacing disturbances—discrete events causing the death of all trees over an area of 0.1 ha or larger—account for 12% of global biomass turnover due to tree mortality. The remaining mortality occurs on a smaller scale, where one or a few trees die while neighbouring trees survive. A solid understanding of mortality at sub‐stand scales is therefore essential for comprehending forest dynamics.

Lightning has long been known to be a small‐scale disturbance in forests (Anderson 1964; Brünig and Brünig 1964; Taylor 1974; Whittaker et al. 1998). However, the detection and adequate quantification of direct (i.e., non‐fire) lightning‐caused tree mortality is highly challenging. Visual signs of lightning strikes are often lacking (or already beyond recognition due to advanced wood decomposition), tree death sometimes occurs slowly, and most reports rely on post hoc surveys or anecdotal evidence of the most conspicuous events (Yanoviak et al. 2017; Zoletto et al. 2023). As a consequence, without prior knowledge of the lightning strike, lightning mortality is often overlooked or erroneously attributed to other causes such as storms, droughts, or biotic agents. A research team recently addressed these issues by combining a camera‐based lightning detection system with subsequent drone and ground‐based field surveys in an old‐growth tropical forest in Barro Colorado Island (BCI), Panama (Yanoviak et al. 2017). They found that cloud‐to‐ground (CG) lightning strikes often do not only affect the directly struck tree but also adjacent trees via “flashovers,” that is, electricity crossing an air gap between neighboring tree crowns. On average, each lightning strike killed 3.5 trees (diameter > 10 cm) within 1 year, including 0.94 large trees (diameter > 60 cm) (Yanoviak et al. 2020). Lightning mortality increased with tree diameter and decreased with distance from the directly struck tree. Comparing lightning mortality rates with background mortality rates in this forest, the authors estimated that lightning is responsible for at least 4.5% of total tree mortality and 40.5% of large tree mortality. Although the former number is confirmed by a previous study conducted in Central Amazonia (Fontes et al. 2018), the authors call these numbers conservative as the assumed lightning density might be underestimated, trees killed from lightning‐crushed trees were not counted as killed by lightning, and some lightning‐damaged trees will only die in the long term (increasing the estimated contribution to large tree mortality to 50.3%). Extrapolating these findings to all tropical forests using local lightning densities, Gora et al. (2020) estimated that lightning annually kills 194 million trees > 1 cm in diameter, including 126 million > 10 cm. Furthermore, lightning is also a major disturbance in other forest types. In some temperate conifer forests, lightning is estimated to be the dominant cause of mortality (Outcalt 2008; Palik and Pederson 1996; Platt et al. 1988), and may kill up to 4.7% of the largest trees each year (Yanoviak et al. 2015). Nevertheless, no quantification about the global ecological importance of lightning mortality currently exists.

Dynamic global vegetation models (DGVMs) are tools that are widely used to simulate terrestrial ecosystems under prescribed environmental conditions. They do so by simulating processes such as tree establishment, growth, competition, and mortality. However, even though DGVMs are constantly refined, their representation of tree mortality is still relatively rudimentary. In many models, wildfire is the only disturbance explicitly simulated, sometimes taking lightning as an ignition source into account (Felsberg et al. 2018; Krause et al. 2014; Rabin et al. 2017). Despite recent research revealing the devastating impacts of lightning strikes on trees, to our knowledge, no DGVM or similar model represents lightning‐caused tree mortality (Veraverbeke et al. 2025). The absence of lightning mortality in DGVMs may cause biases in simulated vegetation dynamics and carbon stocks and challenges the reliability of future simulations, given that most climate models project lightning activity to increase in a warmer world, albeit with considerable uncertainty (e.g., Clark et al. 2017; Finney et al. 2018; Krause et al. 2014). Therefore, representing lightning mortality in DGVMs is crucial to improve their accuracy and usefulness in simulating vegetation response to environmental change.

Here, we address this issue by implementing lightning mortality in the well‐established DGVM LPJ‐GUESS (Smith et al. 2014) to investigate the ecological impacts of lightning at local and global scales. With our simulations, we want to answer the following research questions: (1) Can LPJ‐GUESS reproduce observed lightning‐induced tree mortality in the BCI forest in Panama and other forest sites? (2) How many trees are annually killed by lightning globally? (3) How important is lightning in comparison to other causes of tree mortality in different regions? (4) How does lightning affect forest carbon storage, structure, and composition globally?

## 2 Materials and Methods

### 2.1 The LPJ‐GUESS Model

The LPJ‐GUESS DGVM simulates vegetation dynamics and associated carbon, nitrogen, and water fluxes according to environmental conditions (Smith et al. 2014). It is frequently used for various purposes, for example, for estimating the terrestrial carbon sink (Friedlingstein et al. 2023), quantifying the global impacts of forest disturbances (Pugh et al. 2019), optimizing forest management strategies (Gregor et al. 2024), and isolating the contributions of individual environmental drivers to terrestrial carbon uptake (Krause et al. 2020). LPJ‐GUESS represents an excellent tool to explore the impacts of lightning on forest ecosystems at large spatial scales due to its regional‐to‐global applicability and its within‐stand representation of forest structure and population dynamics. The model is driven by daily surface temperature, short‐wave radiation, precipitation, wind speed, relative humidity, annual atmospheric CO_2_ concentration, and nitrogen deposition. Vegetation diversity is represented by 12 plant functional types (PFTs) which distinguish groups of plants based on characteristics such as growth form, light demand, and bioclimatic limits. Woody PFTs are represented by age cohorts, with all individuals of a cohort sharing the same properties. For each grid cell, a number of replicate patches is simulated to account for vegetation heterogeneity within the grid cell, which is induced by stochastic processes related to tree establishment and mortality. The main mortality mechanisms are background mortality, growth efficiency mortality, wildfires, and patch‐destroying disturbances. Background mortality is calculated from a PFT‐specific longevity, with mortality probability increasing with cohort age (see Figure S1). Growth efficiency mortality occurs if the 5‐year mean growth efficiency of a cohort falls below a PFT‐specific threshold and is particularly relevant for young cohorts competing for light. Wildfire mortality is computed using the SIMFIRE‐BLAZE module, which translates fireline intensities (estimated from fuel load and fire weather parameters) into survival probabilities, taking into account tree size and biome type (Rabin et al. 2017; Nieradzik et al., in preparation). Generic disturbances represent large‐scale mortality events (e.g., windthrows) that occur stochastically with a prescribed probability, resulting in the complete loss of vegetation within the affected patch. Unburned dead biomass is transferred to the litter, where it decomposes according to the CENTURY model (Parton et al. 1993). The organic nitrogen leaves the system via leaching, volatilization by wildfires, or as gaseous soil emissions, or is mineralized and taken up again by plants.

### 2.2 Observed Lightning Mortality in the BCI Forest

**FIGURE 1:**
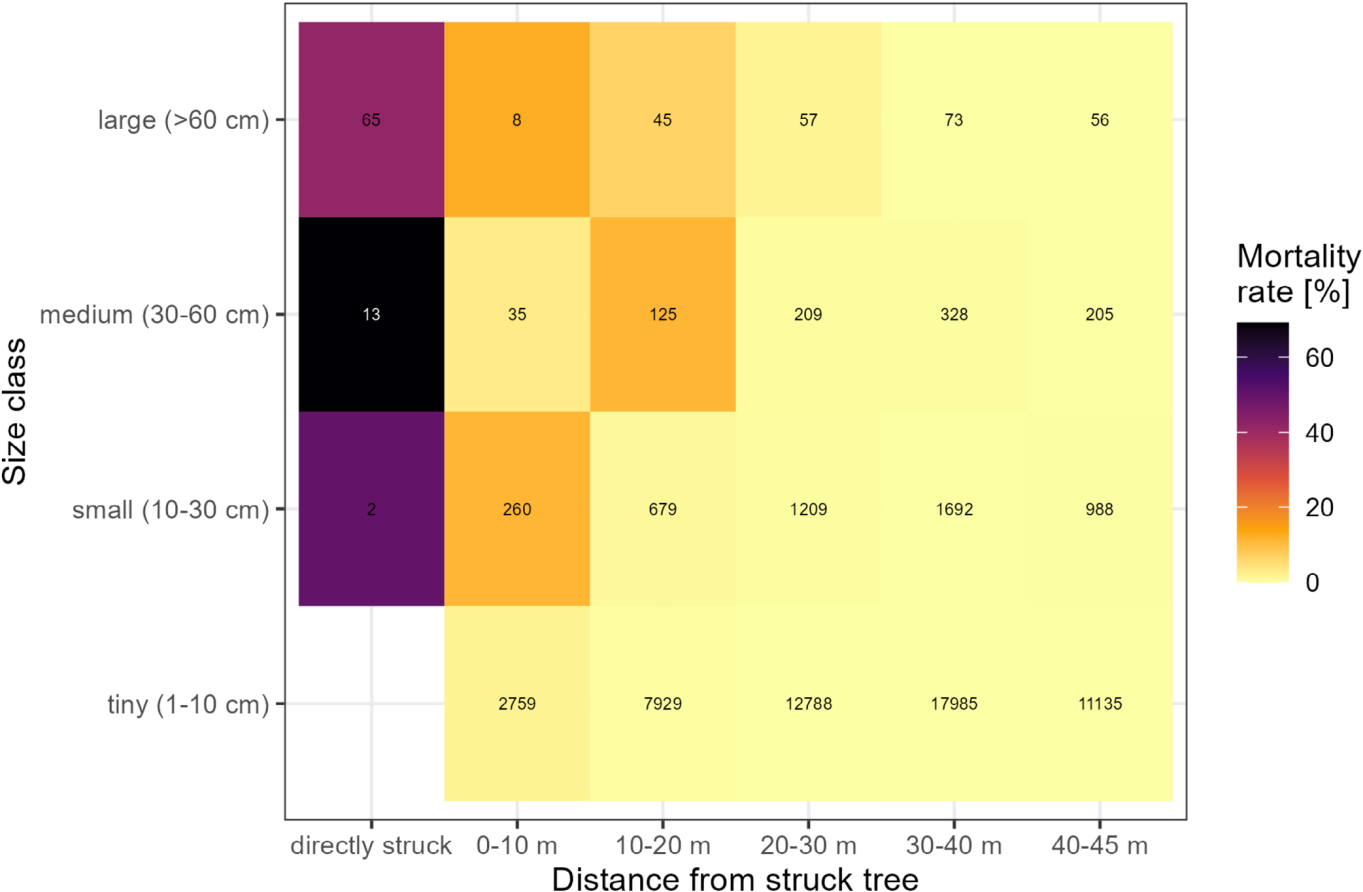
Average lightning‐induced tree mortality rates for different size classes and distances from the strike location from the BCI forest. The number shows the total number of trees per size class and distance zone.

In the BCI forest in Panama, lightning strikes that hit a tree often spread to neighbouring trees and can reach trees up to 45 m away from the struck tree (Yanoviak et al. 2020). Consequently, a single lightning strike often results in the death of multiple trees over the course of several months. To increase the robustness of our implementation, we here applied an updated dataset by Richards et al. (2022) consisting of 97 lightning strikes that occurred during the wet seasons of 2015–2020. Compared to the dataset of Yanoviak et al. (2020) it has the advantage to (1) be based on additional lightning strikes, (2) explicitly report mortality rates of the directly struck trees, and (3) also include tiny trees with a diameter between 1 and 10 cm. As the dataset does not include trees not affected by lightning, we selected all lightning strikes within the BCI forest (for which data about each individual exists from plot surveys) for which the location could be unambiguously identified in the plot and which were surveyed at least 346 days post‐strike (23 strikes) to compute the mortality rates of neighbouring trees. For the mortality rates of directly struck trees, we also included strikes outside of the BCI forest plot (79 strikes). For strikes with multiple surveys after 346 days, we selected the earliest survey (the mean of the earliest surveys was 422 days after the strike). We then computed the average tree mortality rate per strike for different size classes (large trees: > 60 cm diameter; medium trees: 30–60 cm; small trees: 10–30 cm; tiny trees: 1–10 cm) and distances from the strike location (directly struck, 0–10 m, 10–20 m, 20–30 m, 30–40 m, 40–45 m). We computed the mortality rates by dividing the number of trees killed by lightning by the number of living trees within the distance zones according to the plot survey from 2015 (Condit et al. 2019). Overall, lightning impacts were somewhat lower compared to the findings by Yanoviak et al. (2020), for example, only 3.2 trees > 10 cm were killed per strike compared to 3.5 in Yanoviak et al. (2020). However, the updated dataset confirms that mortality rates increase with tree diameter and decrease with distance from the struck tree (Figure 1).

### 2.3 Implementation of Lightning Mortality in LPJ‐GUESS

**FIGURE 2:**
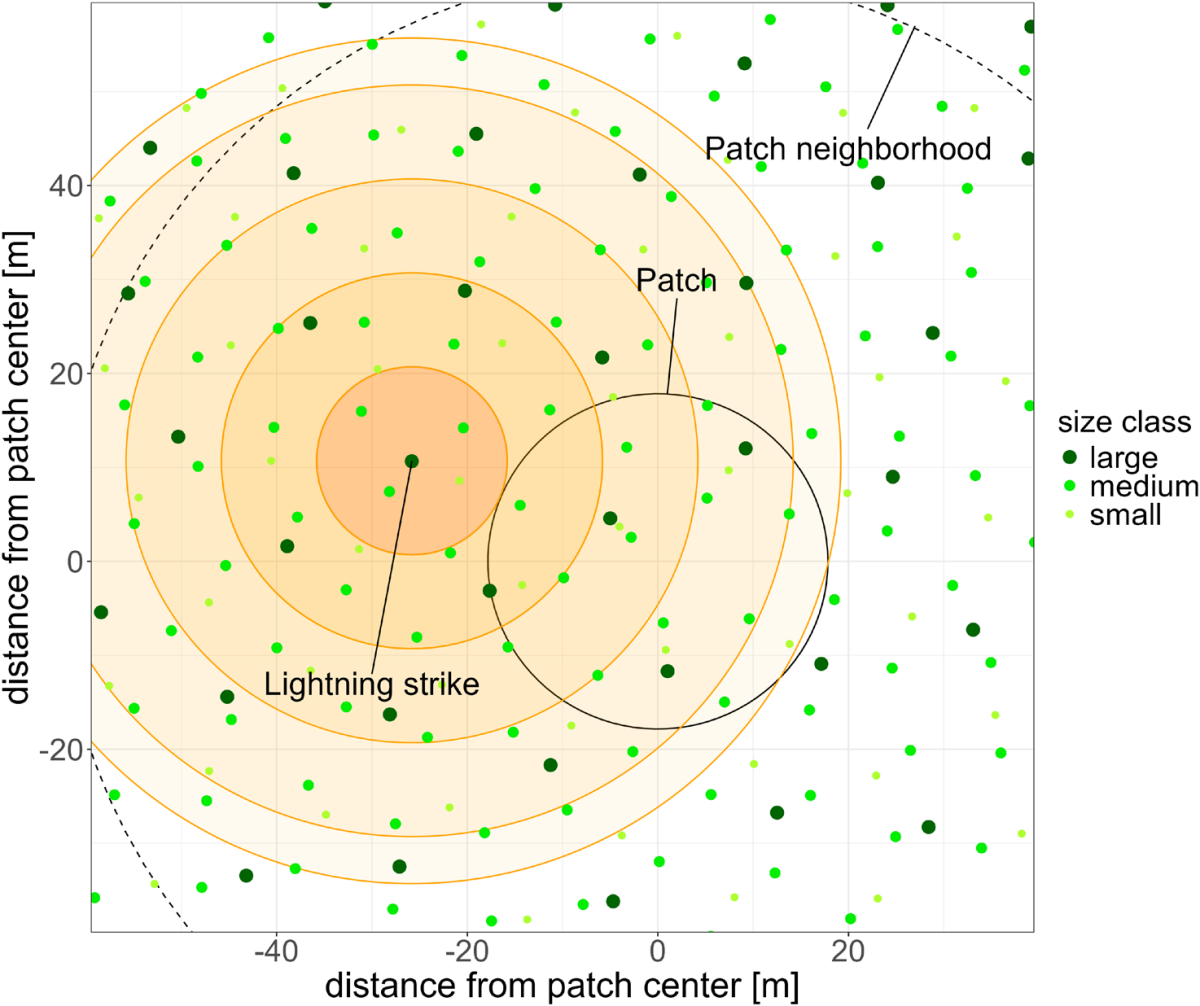
Schematic representation of lightning strikes in LPJ‐GUESS. A strike can kill trees up to 45 m away from the strike location (Richards et al. 2022; Yanoviak et al. 2020). We thus consider not only strikes within a patch but also strikes in the neighbourhood of the patch which are still near enough to kill trees within the patch. In this example, the tree density is 0 for tiny trees, 0.006 individuals per m^2^ for small trees, 0.012 for medium trees, and 0.004 for large trees.

Lightning mortality was implemented at the patch level. The first step in this process was determining how many lightning strikes reached a patch in a given year. To this end, we used binomial probability to calculate the number of lightning strikes hitting a patch based on the prescribed lightning density of the grid cell. If agriculture existed in a grid cell, we assumed equal lightning densities over the natural (i.e., tree‐covered) and agricultural land, although agriculture itself was not affected by lightning. Each lightning strike can kill not only the directly struck tree but also neighboring trees. We therefore did not only consider lightning strikes inside the patch (area 1000 m^2^) but also strikes within the neighborhood that have the potential to kill trees within the patch (i.e., up to 45 m from the patch edge) (Figure 2). Although this increased the target area for lightning strikes from 1000 to 12,406 m^2^ (radius increases from 17.8 to 62.8 m), patches are still only rarely affected by lightning even in regions with high lightning densities (e.g., for the 12.7 flashes yr.^−1^ km^−2^ reported in Panama, the probability for at least one lightning strike hitting a given patch or its neighbourhood is less than 15% each year, see Figure S2a). Additionally, most strikes occur far from the patch center, meaning that the high‐damage zones close to the strike location often lie outside of the patch area (Figure S2b,c). In tropical forests, LPJ‐GUESS tends to generate limited heterogeneity within patches, typically with only a few cohorts/size classes per patch. To address this, we combined five patches for our lightning mortality calculations to represent a more realistic forest structure (Figure S3). This approach assumed that each cohort from the five patches exists in all five patches, but with reduced tree densities. We assumed that each lightning strike that occurred in any of the five patches hits an individual of the tallest cohort across all five patches (which can in principle also be a small or even tiny tree), taking into account that lightning tends to strike the tallest trees (Outcalt 2008; Richards et al. 2022). For the other cohorts, we assumed each strike would also occur on their respective patch but reduced cohort density by a factor of five. For each strike and cohort, we computed the number of individuals in each distance zone from the lightning location by multiplying the reduced cohort density by the area of the respective zone located inside the patch and applied the respective observed mortality rates from the BCI forest (Figure 1). For non‐tropical broadleaf and coniferous PFTs, we assumed a 50% and 80% higher lightning vulnerability, respectively, compared to tropical PFTs (implemented via a PFT‐specific parameter). This assumption is based on their higher electrical resistance (Gora et al. 2017) and the fact that conifers tend to be more prone to lightning than hardwood trees (Baker 1973; Yanoviak et al. 2015).

### 2.4 Global Cloud‐To‐Ground Lightning Densities

**FIGURE 3:**
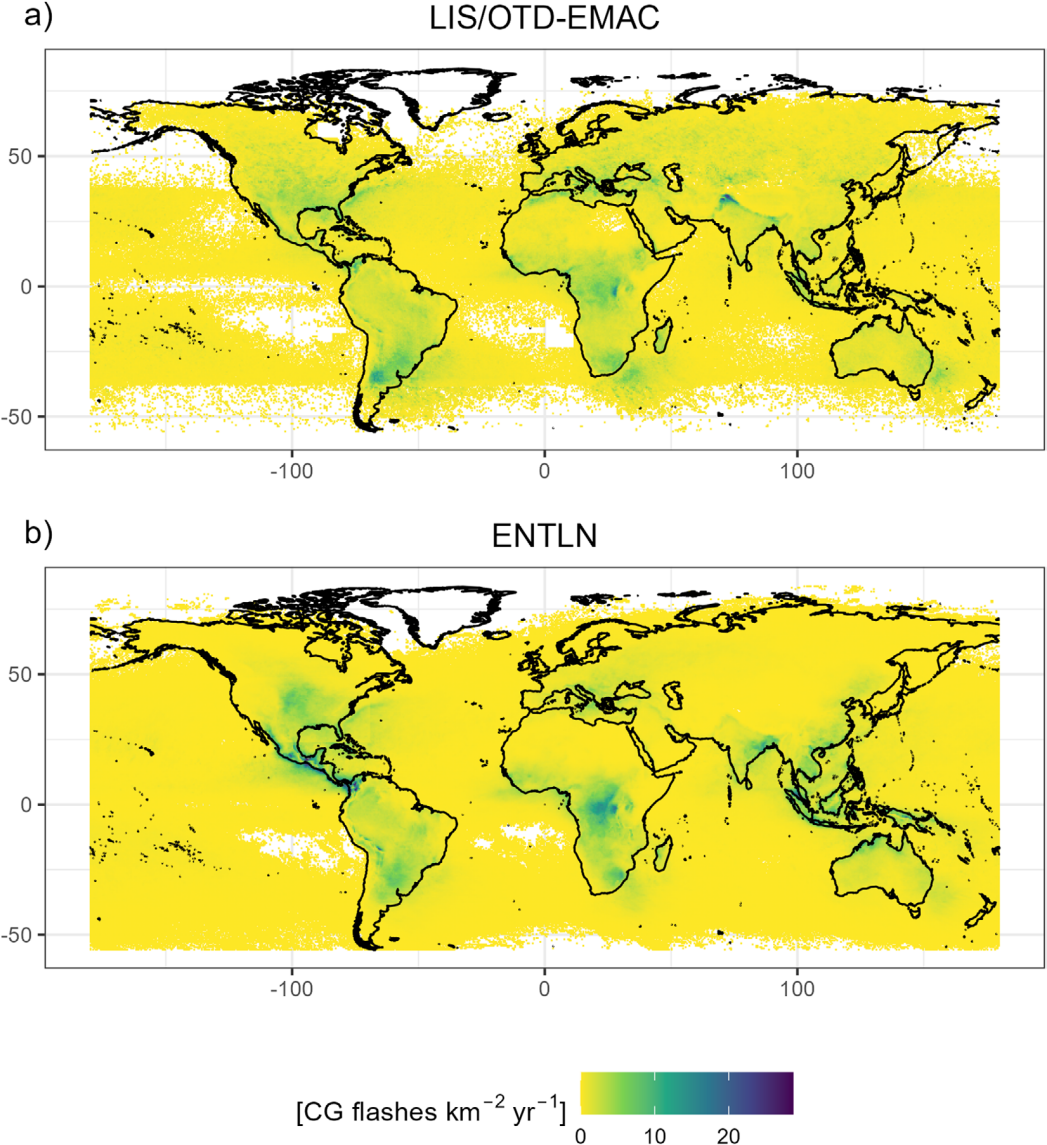
Cloud‐to‐ground lightning densities according to LIS/OTD‐EMAC (i.e., LIS/OTD total lightning densities multiplied by EMAC cloud‐to‐ground fractions) (a) and ENTLN (b).

For the global simulations we accounted for the uncertainty in lightning densities by using two different gridded CG lightning datasets (Figure 3). The first one was derived by multiplying total lightning densities as observed by the space‐born Lightning Imaging Sensor and the Optical Transient Detector (LIS/OTD) (Cecil et al. 2014) with CG fractions as simulated by the chemistry‐climate model EMAC (Pérez‐Invernón et al. 2023, 2022), the latter bilinearly interpolated from the original 2.8° × 2.8° resolution to 0.5° × 0.5° to match the spatial resolution of LIS/OTD. LIS/OTD detect near‐infrared emissions at 777.4‐nm wavelength scattered by lightning through the clouds and emerged from cloud tops. The high‐latitude data comes exclusively from OTD (which was active between 1995 and 2000), while areas within ±38° were also observed by LIS (active between 1998 and 2014). This means that LIS/OTD lightning densities are most robust in the tropics. We used the high‐resolution full climatology (HRFC) (Cecil 2025). According to LIS/OTD, most lightning occurs in the tropics, especially Africa (Figure S4a). However, the tropics are characterized by low CG ratios according to EMAC (Figure S4b). The resulting CG lightning product reveals maxima in central Africa, southern South America and northern India (Figure 3a).

The second dataset was the ground‐based Earth Networks Total Lightning Network (ENTLN) dataset (Zhu et al. 2022), which was provided by Earth Networks (https://www.earthnetworks.com/). This dataset, which also includes observations from the World Wide Lightning Location Network (Rodger et al. 2004), is able to distinguish between CG and intra‐cloud lightning flashes. We constructed a gridded 0.5° × 0.5° product by counting all CG flashes that occurred inside a grid‐cell and dividing by grid‐cell area. ENTLN data was available for the 01/2012–05/2021 period; however, the capture efficiency of ENTLN increased over time due to an increasing number of sensors. As lightning frequency over land showed no obvious trend between 06/2015 and 05/2021 (Figure S5), we assumed no major change in detection efficiency since then and computed the climatology over this time period. Overall, both products have a comparable annual global lightning frequency (286 million vs. 328 million) and a similar lightning distribution (*r* = 0.68), with ENTLN having more lightning in the tropics and LIS/OTD‐EMAC having more lightning in mid‐high latitudes (Figure 3a+b).

### 2.5 Model Evaluation

We compared our simulation results with the observed size distribution of trees directly hit by lightning, the observed number of trees on average killed per lightning strike, and the estimated contribution of lightning to total tree mortality in Panama (Richards et al. 2022). For the size distribution of trees directly hit by lightning, we considered all lightning strikes in the dataset that had information on tree diameter (*n* = 96). For the average number of trees killed by lightning, we included all directly hit trees (*n* = 79) but for neighboring trees, we only included strikes from the BCI forest (*n* = 23) to be consistent with our calculated mortality rates. For the contribution of lightning to total tree mortality, we scaled the estimated contribution of Yanoviak et al. (2020) by the updated number of trees killed per strike (Richards et al. 2022) divided by the original number of trees killed per strike (Yanoviak et al. 2020). For additional model evaluation, we also compared our BCI simulations to estimates on the contribution of lightning to total killed woody biomass (Gora et al. 2021), forest biomass (Chave et al. 2003), net primary productivity (Condit et al. 2013), tree densities of different size classes, and the annual mortality per size class in this forest (Condit et al. 2019).

Furthermore, we also evaluated simulated lightning impacts in other forests for which estimates on lightning mortality exist (Table S1). To this end, we compared the simulated average number of trees killed per lightning strike to estimates for sites in the Peruvian Amazon (Gora and Yanoviak 2020), Brazilian Central Amazon (Magnusson et al. 1996), and Uganda (Zoletto et al. 2023). Furthermore, we compared LPJ‐GUESS simulations to the estimated fraction of large trees annually killed by lightning in the Peruvian Amazon (Gora and Yanoviak 2020) and to the estimated annual lightning mortality and the contribution of lightning to total tree mortality for another site in the Brazilian Central Amazon (Fontes et al. 2018). To assess the applicability of our lightning mortality implementation for extratropical forests, we also compared simulated lightning impacts to estimates from several forest sites in the US: Florida and South Carolina (Outcalt 2008), Michigan (Yanoviak et al. 2015), California (Das et al. 2016), three forests in Texas (Conner et al. 1991), and two forests in Georgia (Palik and Pederson 1996; Platt et al. 1988).

### 2.6 Simulation Setup

We ran LPJ‐GUESS for the BCI forest, the three Amazon sites, and the US sites to evaluate our implementation at the site level. For the BCI forest, we also conducted three sensitivity experiments in which we (a) merged 10 instead of five patches for the lightning mortality calculation; (b) did not merge any patches; (c) did not include lightning mortality. For all site experiments, we ran single 0.5° × 0.5° grid cells under potential natural vegetation with 1000 patches (5000 for the BCI site) to reduce the influence of stochasticity arising from the fact that lightning strikes rarely hit a patch. Cloud‐to‐ground lightning density was taken from the values reported in the studies or—if not provided—from ENTLN, using the lightning density from the 0.1° × 0.1° cell around the site coordinates (see Table S1). We used daily climate forcing from CRU‐JRA‐v2.5 (Harris and Jones 2020; Harris et al. 2020; Kobayashi et al. 2015), atmospheric CO_2_ from the Global Carbon Budget (Friedlingstein et al. 2023), and nitrogen deposition from Lamarque et al. (2013). The total simulation period was 1901–2023, following a 500‐year spin‐up to bring carbon pools to equilibrium. The generic disturbance interval was set to 150 years, the same value as used for the global simulations.

For the global simulations we used the gridded lightning densities derived from either LIS/OTD‐EMAC or ENTLN as input to LPJ‐GUESS. We also conducted a global simulation without lightning. Input climate, atmospheric CO_2_, and nitrogen deposition were from the same datasets as used for the local simulations. As large parts of the globe are managed by humans we accounted for historical land‐use changes by applying net land‐use transitions from the Global Carbon Budget (Chini et al. 2021). Primary land was represented by 35 patches, whereas secondary land (i.e., abandoned agricultural land) was represented by five patches for computational reasons. The value of the disturbance interval (150 years) was chosen so that simulated potential terrestrial biomass (805 GtC over the 2000–2023 period) was well within the ~600–1000 GtC range estimated in other studies (Mo et al. 2023). Simulated potential gross primary productivity over the 2000–2023 period was 135 Gt yr.^−1^, slightly lower than the 142 GtC yr.^−1^ recently estimated by Krause et al. (2022).

## 3 Results

### 3.1 BCI Simulations

**FIGURE 4:**
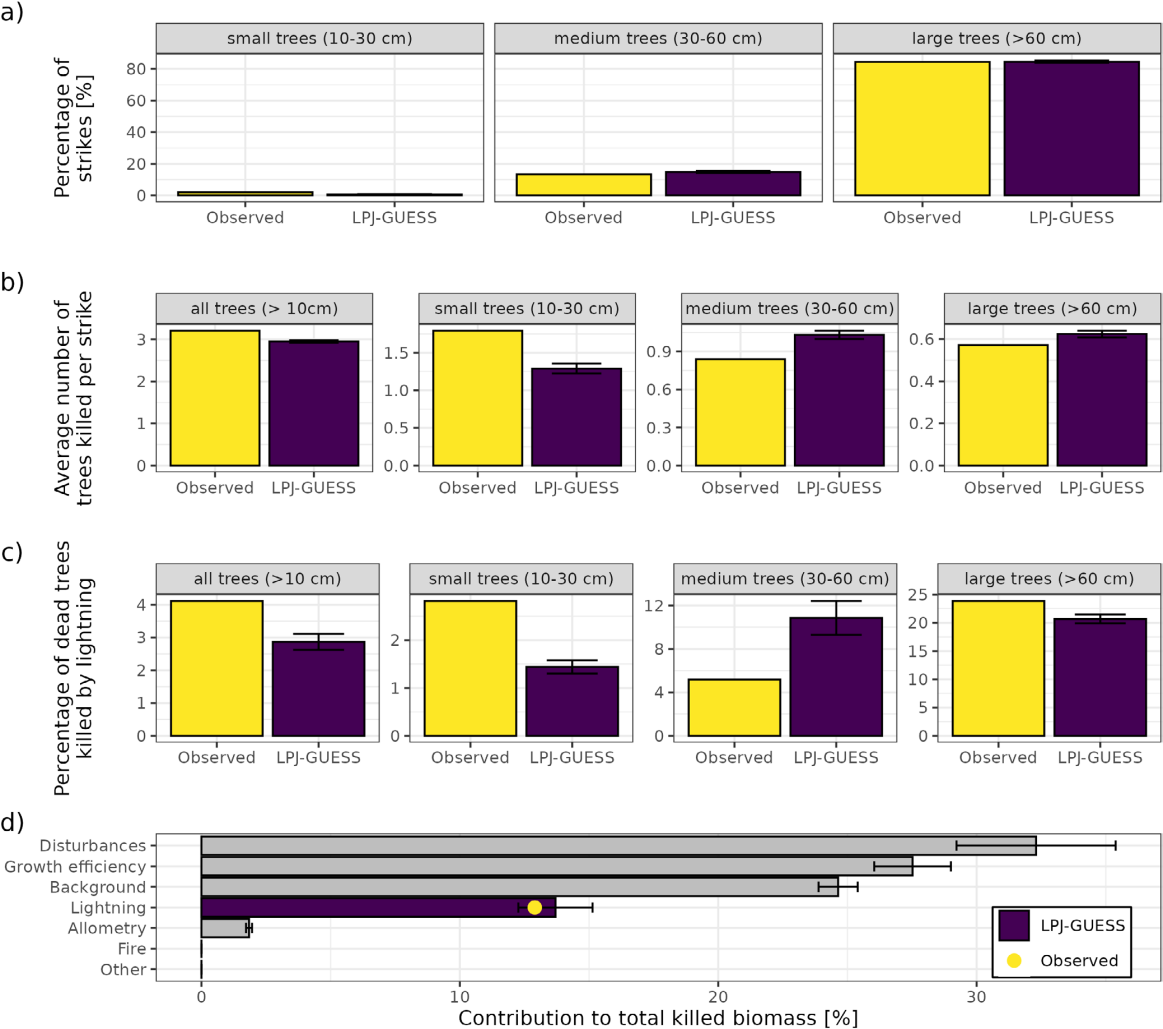
Simulated lightning impacts in the BCI forest compared to the observations from Richards et al. (2022). Proportion of lightning strikes hitting trees of different size classes (a), average number of trees killed per lightning strike for different size classes (b), percentage of dead trees killed by lightning for different size classes (c), and simulated contributions of different mortality causes to total killed biomass (d) averaged over the 2004–2023 period. The error bars represent the variability (1 *σ*) across five simulations, each consisting of 1000 patches.

In the BCI forest, LPJ‐GUESS adequately reproduces observed lightning impacts as well as other forest characteristics such as biomass and tree densities (Figure 4, Figure S6). The model agrees with the observations that around 85% of all lightning strikes hit large trees, with the remaining strikes mostly hitting medium‐sized trees (Figure 4a). It should be noted that this good agreement is largely a result of lightning strikes hitting the tallest cohort of five patches (see Methods). In comparison, hitting the tallest cohort of 10 patches instead leads to 95% of all strikes hitting large trees, whereas hitting the tallest cohort of single patches results in only 33% of lightning strikes hitting large trees (Figure S6). Each lightning strike is simulated to kill on average 2.9 trees (diameter > 10 cm), compared to 3.2 trees in the observations (Figure 4b). The number of small trees killed per strike is underestimated (1.3 vs. 1.8) while the number of medium‐sized trees is slightly overestimated (1.0 vs. 0.84) and the number of large trees is captured well by the model (0.62 vs. 0.57). These patterns are also reflected in the simulated contributions of lightning mortality to the total mortality of trees (Figure 4c): the simulated contribution for small trees is much lower than estimated from observations (1.4% vs. 2.8%), while for medium‐sized trees it is substantially higher (11% vs. 5.2%) and a good agreement for large trees (21% vs. 24%). Overall, lightning is simulated to cause 13.7% of the total killed biomass (Figure 4d), which is similar to the 12.9% contribution to total woody biomass mortality estimated from observations.

At patch level, lightning strikes frequently cause carbon losses, though these losses are generally less severe than those resulting from patch‐destroying disturbances (Figure S7a). The disappearance of the tallest tree cohort is often linked to lightning strikes, with the lost biomass gradually recovering over subsequent decades (Figure S7b). However, in our simulations, lightning mortality typically has limited effects on the amount of light reaching the forest floor, and new cohorts tend to establish only after the most severe events (e.g., the two strikes in 1969/1970 in patches 11–15, see Figure S7c). Instead, the establishment of new cohorts in Panama is more commonly triggered by large‐scale disturbances, especially for shade‐intolerant PFTs.

### 3.2 Other Site Simulations

**FIGURE 5:**
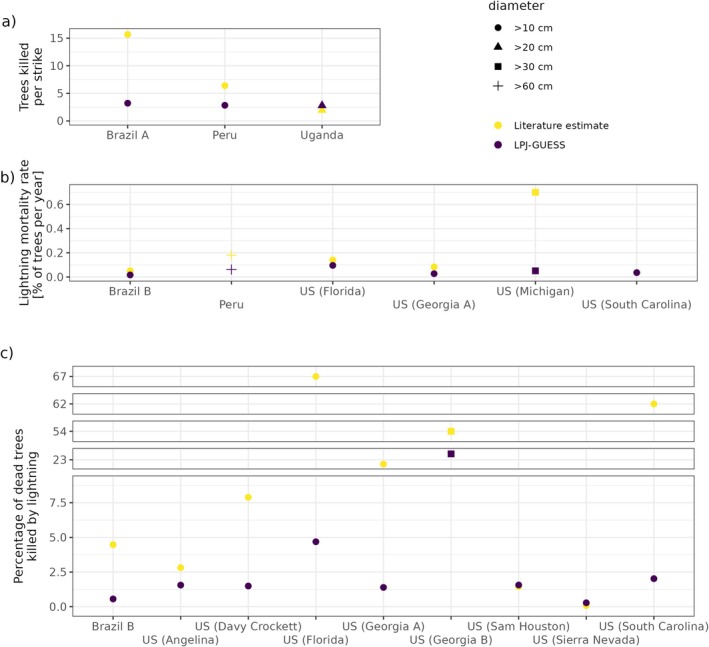
Simulated average number of trees killed per strike (a), percentage of trees annually killed by lightning (b), and percentage of dead trees killed by lightning (c) compared to literature estimates in three Amazonian, one African, and several US forest sites. We assumed trees with unknown mortality cause to not have been killed by lightning. Note that the *y*‐axis is discontinuous in (c).

Comparing our simulation results to estimates on lightning mortality from other forest sites yields large variations in simulated and observed lightning impacts across sites (Figure 5). LPJ‐GUESS simulates substantially lower numbers of trees killed per strike than estimated in two studies in the Amazon, while for the Uganda site the model slightly overestimates the number of killed trees per strike (Figure 5a). Concerning the annual fraction of trees killed by lightning each year, observed impacts are reproduced for some sites in the US (Florida, South Carolina) but underestimated by the model for other sites (Figure 5b). Similarly, the contribution of lightning to the total mortality is roughly captured for some sites (Angelina, Sam Houston, Sierra Nevada) but severely underestimated for other sites for which the observations sometimes suggest contributions of more than 50% (Figure 5c). Interestingly, despite a limited local lightning density, LPJ‐GUESS simulates a much larger contribution for the Georgia B site than for the other sites. However, this can largely be explained by stochasticity as the relevant size classes (> 30 cm diameter) only occur on a small fraction of the patches.

### 3.3 Global Simulations

**FIGURE 6:**
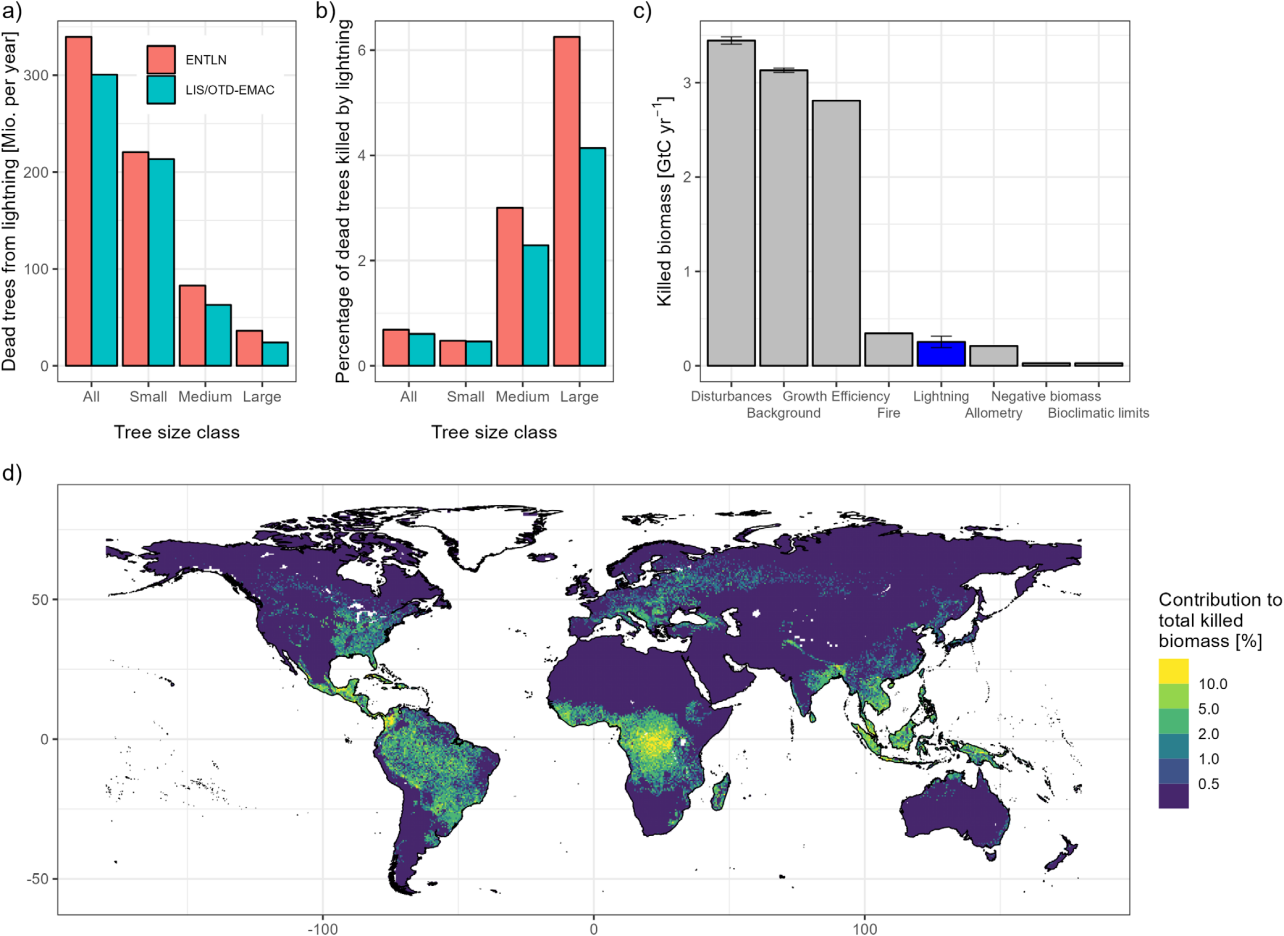
Simulated global lightning mortality. Total number of killed trees (a) and percentage of trees killed by lightning (b) for different size classes, contribution of lightning to total killed biomass (c), and maps of the contribution of lightning to total killed biomass (d). (c, d) show the mean of the LIS/OTD‐EMAC and ENTLN simulations.

Globally, 286–328 million lightning strikes hit the Earth's surface each year, 196–204 million of which occur over ice‐free land areas. According to our simulations, this results in the annual death of 301–340 million trees (diameter > 10 cm) over the 2004–2023 period (160–234 million in the tropics within ±23.5°), including 24–36 million large trees (Figure 6a). For comparison, around 50 billion trees are annually killed by natural causes, including 581 million large trees. Although lightning is thus responsible for 0.61%–0.69% of all tree death, this fraction is much higher for large trees (4.1%–6.3%, Figure 6b). This is not only because of the higher susceptibility of large trees to lightning but also because large trees are simulated to occur mostly in the tropics where lightning densities are high. Overall, lightning is responsible for a dead biomass of 0.21–0.30 GtC yr.^−1^, corresponding to 2.1%–2.9% of the total biomass killed by natural causes (Figure 6c). Interestingly, this amount is comparable to the 0.34 GtC annually lost due to wildfires (excluding litter combustion). The largest contribution of lightning to total killed biomass is found in Central Africa (Figure 6d), consistent with the lightning hotspot in this region (Figure 3).

Comparing these global simulations with a simulation without lightning mortality, lightning turns out to have small but noticeable impacts on vegetation carbon storage and structure (Figure S8). Concerning total living biomass, the presence of lightning reduces present‐day vegetation carbon by 6.9–9.3 GtC, equivalent to a reduction of 1.3%–1.7%. This is largely due to a reduction in broadleaved biomass, while conifers, despite their higher lightning vulnerability, are hardly affected by lightning due to the low lightning densities in the boreal zone. Lightning slightly increases the number of small trees but reduces the number of medium‐sized and, in particular, the number of large trees (by 2.3%–3.0% or 882/1143 million trees).

## 4 Discussion

### 4.1 Lightning Impacts

In this study, we implemented a lightning‐caused tree mortality module into the LPJ‐GUESS model to investigate the direct impacts of lightning strikes (i.e., not by wildfire ignitions) on forest ecosystems. Aligning with the limited number of observations, our simulations suggest that lightning is an important cause of tree mortality in many regions, causing 0.21–0.30 GtC of dead biomass each year at a global scale. The largest impacts were simulated in tropical Africa, where lightning density is high and large trees, which are most vulnerable to lightning, prevail. The simulated number of tropical trees killed by lightning (160–234 million) is higher than the 126 million trees estimated by Gora et al. (2020) who also used ENTLN lightning densities (2013–2018 though) and lightning mortality data from Yanoviak et al. (2020) but excluded savannahs and shrublands. Indeed, the simulated lightning impacts in our study may be underestimated for two reasons: (1) LPJ‐GUESS tends to simulate lower lightning mortalities than estimated in other tropical and temperate forests (even though some estimates might be biased towards high lightning mortality, for example, by looking only at forest gaps created by lightning); (2) we only consider mortality directly associated with lightning strikes but neither growth reductions (Camarero et al. 2020) nor long‐term premature mortality (Yanoviak et al. 2020) or secondary biotic mortality of trees damaged but not directly killed by lightning. On the other hand, the number of trees killed per strike is higher when only considering neighboring trees from inside the BCI forest (which we did to compute lightning mortality rates) than when including killed trees from all strikes (3.2 vs. 2.6). This may indicate that strikes inside the BCI forest were, by chance, more destructive than a typical strike in the region (and thus our lightning mortality rates too high) but could also be a result of sampling biases or higher tree densities compared to nearby forests. Overall, our results can be regarded as a first rough estimate of the global ecological importance of lightning, indicating that ecosystem models should account for lightning mortality to more reliably simulate forest dynamics and terrestrial carbon cycling.

### 4.2 Applicability of Our Implementation at Global Scale and Ways Forward

Our implementation of lightning‐induced tree mortality in LPJ‐GUESS adequately reproduces observed lightning impacts in the BCI forest, especially for large trees which store substantial amounts of carbon (Figure 4). Deviations between model results and observation‐based estimates for small and medium‐sized trees for this site are likely partly related to how the other mortality mechanisms in LPJ‐GUESS work: small trees have a far higher mortality rate than in the observations because of high growth efficiency mortality under competition for light, but once trees reach their “middle‐age” lifespan, their mortality is lower than in the observations (Figure S6). For large trees which are typically 150–300 years old (Figure S9), background mortality becomes increasingly important as they mature (see Figure S1). This is likely the reason why no giant trees > 100 cm in diameter are simulated by the model, even when switching off lightning mortality (Figure S6), while in reality these trees make up 17% of the total biomass (Chave et al. 2003).

Concerning the transferability to other forest types, Yanoviak et al. (2020) reasoned that lightning is a major cause of tree mortality in various tropical forests. However, Gora et al. (2023) highlighted that the severity of lightning damage may vary depending on forest complexity, such as the presence of lianas (which are not represented in LPJ‐GUESS). Additionally, Zoletto et al. (2023) reported a lower mortality rate (7 out of 20) among trees directly struck by lightning in Uganda, and the simulated number of trees killed per strike was slightly higher than observed (Figure 5a). On the other hand, LPJ‐GUESS simulates a lower number of trees killed per strike than reported in Brazil and Peru. However, it is likely that these gap‐focused studies only detected the more severe lightning strikes and thus overestimated the average impact of a strike (Gora and Yanoviak 2020). We thus assume that our lightning mortality module can also be applied elsewhere in the tropics even though more observational data especially from the African rainforest would be highly valuable to confirm the high simulated lightning impacts in this region.

For temperate and boreal forests, no systematic studies comparable to Yanoviak et al. (2020) and Richards et al. (2022) exist. Instead, estimates on lightning mortality in these regions are typically based on post hoc surveys conducted at infrequent intervals. One pattern observed in both temperate and tropical forests is large trees being more often hit by lightning than smaller trees (e.g., Outcalt 2008; Palik and Pederson 1996; Platt et al. 1988), suggesting that at least this aspect of our implementation is also suitable for extratropical forests. Unfortunately, most of the temperate forest studies were conducted in pine forests in the US, potentially because lightning damage to pines is easily recognized due to its characteristic longitudinal scars (Baker 1973). The issue is further complicated by the fact that lightning‐damaged conifers are often colonized by bark beetles (e.g., Conner et al. 1991; Outcalt 2008), making it difficult to attribute tree death to a single mortality agent. Consequently, a comparison of estimated versus simulated lightning mortality is challenging in these forests and the applicability of our implementation more uncertain than in the tropics. Although the lower‐than‐estimated simulated lightning mortality in several US forests (Figure 5b+c) suggests that our approach might underestimate the vulnerability of extratropical forests to lightning, it does not necessarily imply poor model performance. Deviations from field observations might be caused by survey errors (due to the challenges mentioned in the introduction), exaggerations (e.g., for the Michigan forest lightning mortality apparently was set equal to lightning damage), small sample size or stochasticity (e.g., the Brazilian B estimate was derived from a single year of observations in which one lightning event was documented that killed three trees), differences in forest structure (e.g., average observed tree diameter was 34 cm in Florida while no trees > 30 cm were simulated by LPJ‐GUESS), or erroneous local lightning densities. Additionally, LPJ‐GUESS simulates broadleaved PFTs (which have a lower lightning susceptibility than conifers) to outperform needleleaved PFTs in some of the US sites, while in reality most of these forests are dominated by pine species. These forests might thus not be captured well by our global PFT approach.

In any case, more field observations, especially from non‐pine forests and from regions outside the US, are needed to improve the representation of lightning mortality in DGVMs or Earth System Models for global applications (Veraverbeke et al. 2025). Ideally, these would involve systematic lightning monitoring systems as in Yanoviak et al. (2017) but could be complemented by post hoc surveys using drones (Gora and Yanoviak 2020) or regular ground surveys. Real‐time lightning monitoring would be particularly important to accurately assess the number of killed trees and the typical area of effect of a lightning strike in different forest types. Although single tree deaths per strike seem to be common in temperate forests, group mortality events have also been reported (e.g., Outcalt 2008; Palik and Pederson 1996) but it needs to be quantified how common these really are. The importance of environmental factors, such as soil moisture, terrain, or peak flash current, on lightning damage also requires more investigation (Mäkelä et al. 2009). For instance, individual strikes tend to cause more damage in valleys than on ridges, possibly because the higher lightning frequency on ridges promotes the survival of lightning‐tolerant tree species (Gora et al. 2025). In temperate and boreal forests, the timing of lightning strikes during the season may also play a role, as trees struck during cooler periods tend to have a higher survival rate (Baker 1973). Moreover, gaining more knowledge about the lightning susceptibility of different tree species and the influence of tree vitality—potentially weakened by more frequent droughts—is needed to reliably project changes in forest composition under changing lightning activity. Existing studies already suggest that conifers are more likely to be struck and/or more prone to lightning than broadleaved trees (Yanoviak et al. 2015) and species with denser wood tend to be more tolerant of lightning strikes (Richards et al. 2022). These patterns are somewhat reflected in our lightning vulnerability parameters of different PFTs, but poorly constrained quantitatively by empirical evidence. Additionally, there are known differences between species that are grouped into one PFT in LPJ‐GUESS. For instance, oak seems to be more often struck by lightning than beech and pine more often than spruce (Covert 1924). However, such differences cannot be captured by our global PFT approach and would require the representation of actual tree species as, for example, possible in the European version of LPJ‐GUESS (Lindeskog et al. 2021).

### 4.3 Uncertainties in Global Lightning Activity

Another key uncertainty in quantifying the global impacts of lightning on terrestrial ecosystems is limited knowledge about how much lightning occurs in different regions around the globe. We address this uncertainty by using two gridded products of CG lightning. The first one (LIS/OTD‐EMAC) is a combination of satellite‐derived total lightning densities with CG fractions from a chemistry‐climate model. Notably, LIS/OTD is planned to be merged with its “successor” ISS LIS (Blakeslee et al. 2020), which will make lightning densities more robust in mid‐high latitudes. Nevertheless, having to convert total lightning to CG lightning will remain a source of uncertainty. Although climate models generally agree on CG fractions increasing with latitude (e.g., Krause et al. 2014), simulated global spatial patterns need to be confirmed via independent estimates as available for some regions (e.g., Boccippio et al. 2001). The lower lightning densities in LIS/OTD‐EMAC compared to ENTLN in the tropics suggest that EMAC might underestimate the CG fraction in this region. The ground‐based ENTLN product thus represents a valuable alternative lightning input as it can distinguish between CG and cloud‐to‐cloud lightning. It has the disadvantage that its detection efficiency depends on the sensor density and may thus differ across space and time. However, global lightning frequency in this dataset was relatively stable over our selected period (2015–2021) and slightly higher than for LIS/OTD‐EMAC, suggesting that ENTLN captures most CG strikes.

Additionally, lightning impacts are sensitive to changes in lightning frequency over time. We here applied lightning climatologies over the entire simulation period because the time series products of LIS/OTD and ENTLN only cover a few years and for LIS/OTD show no obvious trend (Cecil et al. 2014). However, there is some evidence that thunderstorm activity has increased in several regions around the world over the last decades (Harel and Price 2020; Kaplan and Lau 2021; Simon et al. 2023) and is expected to further increase in a warmer world (Clark et al. 2017; Krause et al. 2014; Pérez‐Invernón et al. 2023), at least in extratropical regions (Chen et al. 2021; Finney et al. 2018; Romps et al. 2014). This might increase lightning‐induced mortality, shift forest composition and structure, and reduce overall vegetation carbon storage. Accordingly, Yanoviak et al. (2020) estimate a 9%–18% increase in large tree mortality for a 25%–50% increase in lightning frequency based on simple upscaling of their findings. However, this approach does not account for the concurrent impacts and interplay of changes in other environmental drivers. A consequent next step is to run the lightning‐enabled LPJ‐GUESS version with consistent scenarios of climate change, atmospheric CO_2_, nitrogen deposition, land‐use change, and associated changes in lightning frequency to obtain a more accurate perspective of how future forests might look like in the face of global environmental change and to better quantify the risk that the land carbon sink might turn into a carbon source.

### 4.4 Other Disturbances

In this study we focus on the direct impacts of lightning strikes on trees. However, lightning can influence terrestrial ecosystems in manifold ways. The importance of lightning as a wildfire ignition source is relatively well understood (e.g., Menezes et al. 2022) and already included in some ecosystem models (e.g., Felsberg et al. 2018). However, the currently available fire modules in the standard LPJ‐GUESS version do not account for lightning ignitions (Rabin et al. 2017). This is particularly problematic in remote regions like the boreal forest where lightning‐ignited fires are responsible for most of the total burned area (Janssen et al. 2023). Additionally, lightning‐damaged trees are prone to subsequent disturbances such as windthrows, pathogens, or insect attacks (Johnson 1966; Parlato et al. 2020; Taylor 1974). In this regard, LPJ‐GUESS provides a unique opportunity to study the interactions and combined ecological impacts of various disturbances as the new lightning mortality module could be coupled with existing modules of storm damage (Lagergren et al. 2012), bark beetle outbreaks (Jönsson et al. 2012), wildfires (Rabin et al. 2017), frost damage (Meyer, Buras, et al. 2024), and droughts (Meyer, Darela‐Filho, et al. 2024; Papastefanou et al. 2024).

## 5 Conclusions

Lightning is an important disturbance in forest ecosystems. However, direct (i.e., not fire‐related) lightning mortality is so far often underestimated and consequently neglected in computer models used to study how forest ecosystems respond to environmental changes. By implementing a lightning mortality module into the ecosystem model LPJ‐GUESS, we find that lightning kills around 320 million trees each year, thereby causing ~0.25 GtC of dead biomass. These findings confirm existing studies arguing that lightning mortality is an important cause of tree mortality in many forests. Clearly, there is a need to better understand the ecological impacts of lightning in different forest types. Our simulations provide a first estimate of lightning‐induced tree mortality and associated carbon impacts at a global scale. Nevertheless, it is important to extend field data on lightning‐induced tree mortality and to incorporate lightning mortality into additional vegetation models to confirm our findings.

## Author Contributions

**Andreas Krause:** conceptualization, formal analysis, funding acquisition, investigation, methodology, project administration, software, visualization, writing – original draft. **Konstantin Gregor:** methodology, writing – review and editing. **Benjamin F. Meyer:** methodology, writing – review and editing. **Anja Rammig:** conceptualization, supervision, writing – review and editing.

## Conflicts of Interest

The authors declare no conflicts of interest.

## Supporting information


Data S1

## Data Availability

The LPJ‐GUESS model code and the data that support the findings of this study are openly available in Figshare at https://doi.org/10.6084/m9.figshare.29255384.v1. LIS/OTD lightning data were obtained from NASA at https://doi.org/10.5067/LIS/LIS‐OTD/DATA302. EMAC model output were obtained from Zenodo at https://doi.org/10.5281/zenodo.6627112. ENTLN lightning data are freely available for scientific use from Earth Networks upon request at https://www.earthnetworks.com/. Panama lightning mortality and census data were obtained from Dryad at https://doi.org/10.5061/dryad.gf1vhhmsp and https://doi.org/10.15146/5xcp‐0d46, respectively.
