# Supplementary material for: Simulating Lightning‐Induced Tree Mortality in the Dynamic Global Vegetation Model LPJ‐GUESS
Source: Glob Chang Biol. 2025 Jun 24;31(6):e70312. doi: 10.1111/gcb.70312 (PMC12186139; doi:10.1111/gcb.70312)
Supplement: Supplementary file 1 — Data S1 [file GCB-31-e70312-s001.pdf]

Supporting information for "Simulating lightning-induced tree mortality in the dynamic global vegetation model LPJ-GUESS" by Krause et al. (2025)

Table S1: Overview of studies which estimated lightning mortality.

Impact type	Region (forest type)	Coordinates (LPJ-GUESS grid-cell)	Lightning density [CG flashes km^{-2} yr^{-1}] (source)	Study duration	Number of trees in observations killed by lightning, killed in total, and total number of trees investigated	Size class (observed vs. simulated)	Lightning impact (observed *vs.* simulated)	Notes	Reference
Number of trees killed per lightning strike	Peru (lowland rainforest)	3.252°S, 72.907°W (3.25°S, 72.75°W)	2.1 (reported based on ENTLN; assumed to be a lower bound)	2019	45 trees killed by seven lightning strikes	>10 cm diameter (both)	6.4 vs. 2.84	Gap-focused, thus likely biased toward the larger end of the disturbance severity distribution	(Gora & Yanoviak, 2020)
	Brazil A (rainforest)	3°8'S, 60°4'W (3.25°S, 60.25°W)	2.96 (ENTLN)	1988	47 trees killed in three gaps	>10 cm diameter (both)	15.67 vs. 3.22	Three large gaps, thus likely biased toward the larger end of the disturbance severity distribution	(Magnusson, Lima, & deLima, 1996)
	Uganda (montane tropical forest)	1°3'S, 29°43'W (1.25°S, 29.75°W)	~15 (reported)	2022	122 trees killed at 62 sites	>20 cm diameter (?) vs. >10 cm diameter	1.97 vs. 2.80	Based on lightning scars, park ranger reports, and flashover criteria	(Zoletto, van der Sande, van der Sleen, & Sheil, 2023)
Lightning mortality rate [% of trees per year]	Brazil B (rainforest)	2°37'S, 60°11'W (2.75°S, 60.25°W)	2.05 (ENTLN)	2010-2011	3/67/5808	>10 cm diameter (both)	0.052% vs. 0.017%	Only three trees killed during a single storm	(Fontes, Chambers, & Higuchi, 2018)
	Florida (longleaf pine and occasional oak)	29°10'25"N, 81°49'18"W (29.25°N, 81.75°W)	~11.0 (reported)	1992-1997	236/354/16692	mean diameter 25.6 cm (range 13.5-54 cm for lightning-killed trees but >5 cm for all trees) vs. >10 cm diameter	0.14% vs. 0.096%		(Outcalt, 2008)
	South Carolina (longleaf pine)	33.246°N, 81.668°W (33.25°N, 81.75°W)	~4.0 (reported)	1993-1996	130?/210?/93330 (0.62*0.206*4*255)	mean diameter 23.9 cm vs. >10 cm diameter	0.036% (lightning mortality 0.132 trees ha^{-1} yr^{-1}/tree density 366 trees ha^{-1}) vs. 0.037%		
	Georgia A (longleaf pine)	31.194839°N, 84.468623°W (31.25°N, 84.25°W)	3.11 (ENTLN)	1990-1994	45?/203/-	>10 cm diameter (both)	0.084% vs. 0.028%	48% died from unknown causes, no hurricanes during study period	(Palik & Pederson, 1996)
	Michigan (hemlock-hardwood)	46.87°N, 87.88°W (46.75°N, 87.75°W)	1.2 (reported based on NLDN; 60%–80% detection	2013	14/-/"thousands"	"largest trees" (>30 cm diameter?) vs. >30 cm diameter	0.7% vs. 0.051%	Lightning mortality rate seems to be set equal to lighting	(Yanoviak et al., 2015)

			efficiency)					damage rate (0.7%)	
	Peru (lowland rainforest)	3.252°S, 72.907°W (3.25°S, 72.75°W)	2.1 (reported based on ENTLN; assumed to be a lower bound)	2019	45 trees killed by seven lightning strikes	>60 cm diameter (both)	0.18% vs. 0.062%		(Gora & Yanoviak, 2020)
Percentage of dead trees killed by lightning	Brazil B (rainforest)	2°37'S, 60°11'W (2.75°S, 60.25°W)	2.05 (ENTLN)	2010-2011	3/67/5808	>10 cm diameter (both)	4.5% vs. 0.56%	Only three trees killed during a single storm	(Fontes et al., 2018)
	California (Sierra Nevada; different forest types)	23 plots around 36.6°N, 118.8°W (36.75°N, 118.75°W)	0.432 (ENTLN)	1998-2010	3/3729/23657	>137 cm height vs. >10 cm diameter	0.081% vs. 0.28%	Lightning damage found on three dead trees	(Das, Stephenson, & Davis, 2016)
	Florida (longleaf pine and occasional oak)	29°10'25"N, 81°49'18"W (29.25°N, 81.75°W)	~11.0 (reported)	1992-1997	236/354/16692	mean diameter 25.6 cm (min diameter 5 cm?) vs. >10 cm diameter	67% vs. 4.7%		(Outcalt, 2008)
	South Carolina (longleaf pine)	33.246°N, 81.668°W (33.25°N, 81.75°W)	~4.0 (reported)	1993-1996	130?/210?/93330 (0.62*0.206*4*255)	mean diameter 23.9 cm vs. >10 cm diameter	62% vs. 2.0%		
	Georgia A (longleaf pine)	31.194839°N , 84.468623° W (31.25°N, 84.25°W)	3.11 (ENTLN)	1990-1994	45?/203/-	>10 cm diameter (both)	22% vs. 1.4%	48% died from unknown causes, no hurricanes during study period	(Palik & Pederson, 1996)
	Georgia B (longleaf pine)	30°45'N, 84° 00'W (30.75°N, 84.25°W)	3.66 (ENTLN)	1980-1983	?/?/9340 (>2 cm diameter)	>30 cm diameter vs. > 10 cm diameter (no trees >30 cm were simulated)	54% vs. 24%		(Platt, Evans, & Rathbun, 1988)
	Texas (Angelina; pine)	31°16'N, 94°25'W or 31°16'7"N, 94°24'43"W (31.25°N, 94.25°W)	4.11 (ENTLN)	1978-1990	2/71/-	? vs. >10 cm diameter	2.8% vs. 1.6%	Woodpecker cavity trees only	(Conner, Rudolph, Kulhavy, & Snow, 1991)
	Texas (Davy Crockett; pine)	31°20'00"N 95°05'00"W (31.25°N, 95.25°W)	4.45 (ENTLN)	1987-1989	3/38/-		7.9% vs. 1.5%		
	Texas (Sam Houston; pine)	30°35'04"N 95°07'57"W (30.75°N, 95.25°W)	4.36 (ENTLN)	1983-1988	5/344/-		1.5% vs. 1.6%		

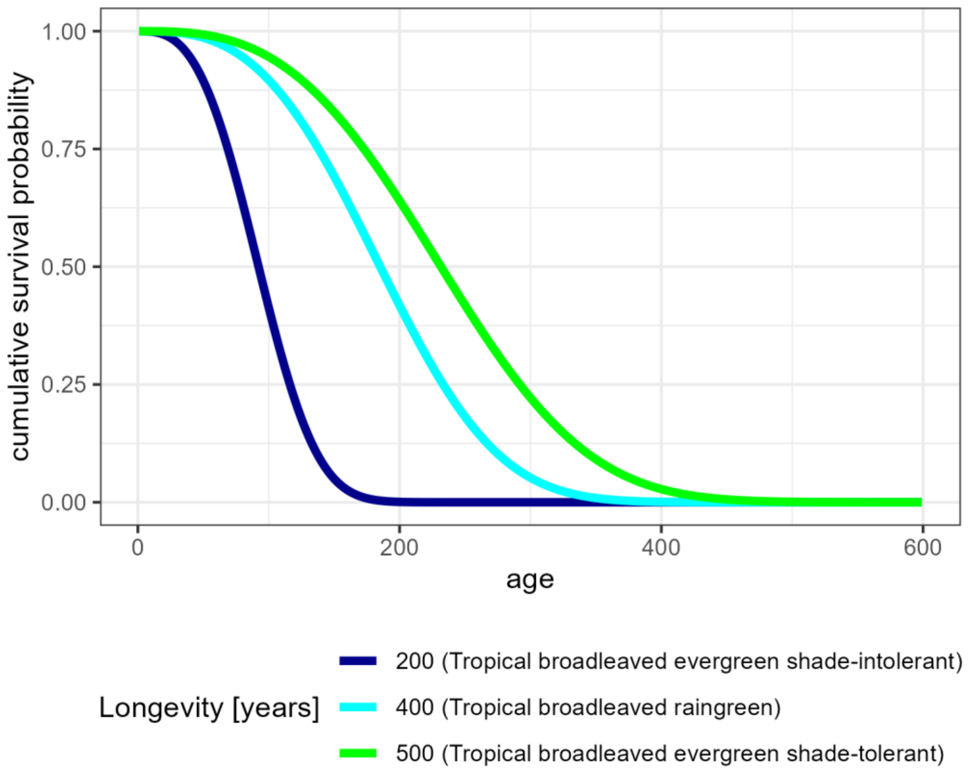

Fig. S1: Cumulative survival probability of different tropical PFTs dependent on cohort age. The tropical broadleaved evergreen shade-tolerant PFT is the dominant simulated PFT in Panama.

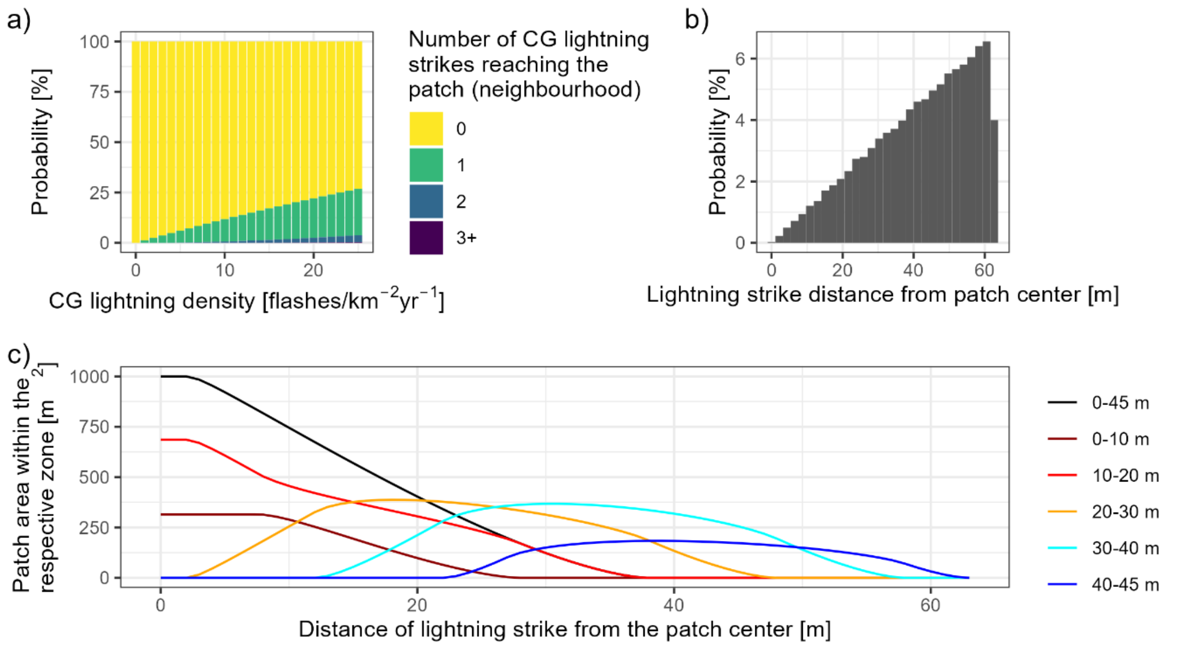

Fig. S2: Lightning statistics. Probability distribution of the number of CG lightning strikes hitting a patch or its neighbourhood (i.e. up to 62.84 m from the patch centre) in a given year as a function of the grid-cell's lightning density (a). Probability distribution of lightning strike distance to the patch centre (b). Patch area lying in different distance zones around the lightning strike location as a function of lightning strike distance from the patch centre (c).

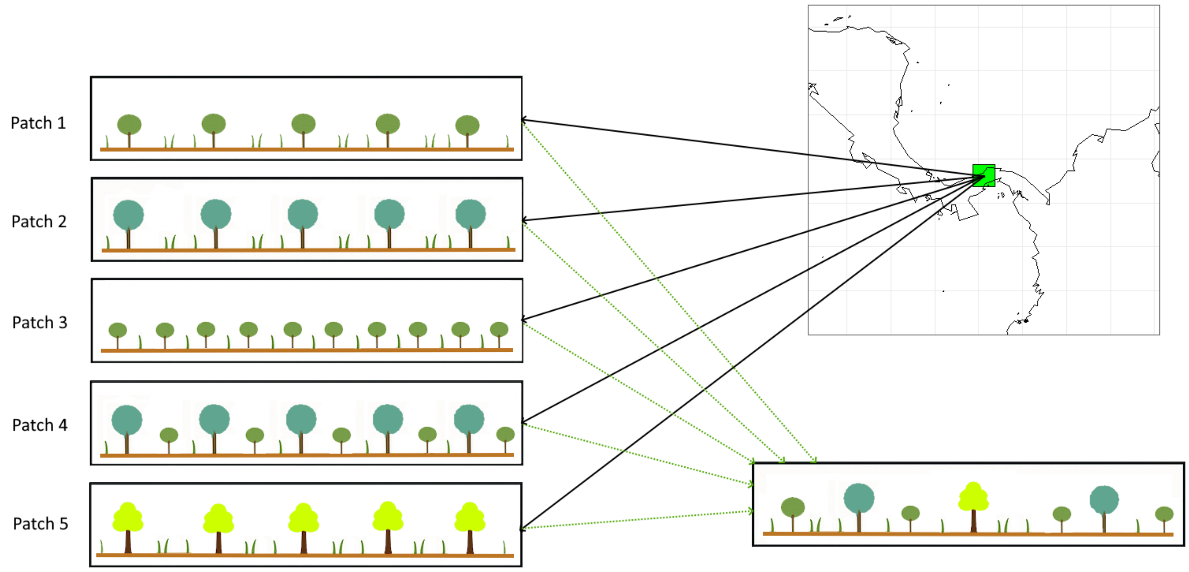

Fig. S3: Merging of patches for the lightning mortality calculation. A grid-cell in LPJ-GUESS (0.5°x0.5°) is represented by multiple patches (here five for simplicity; 1000 for site simulations) which distinguish in terms of forest structure and composition due to stochastic processes related to establishment and mortality. Five patches are merged respectively for the lightning mortality calculation. Lightning strikes hit the tallest cohort across the five patches. The figure was created in paint.net, the map was created in R using the ggplot2 package (Wickham, 2016) and the tree shapes were designed by ChatGPT.

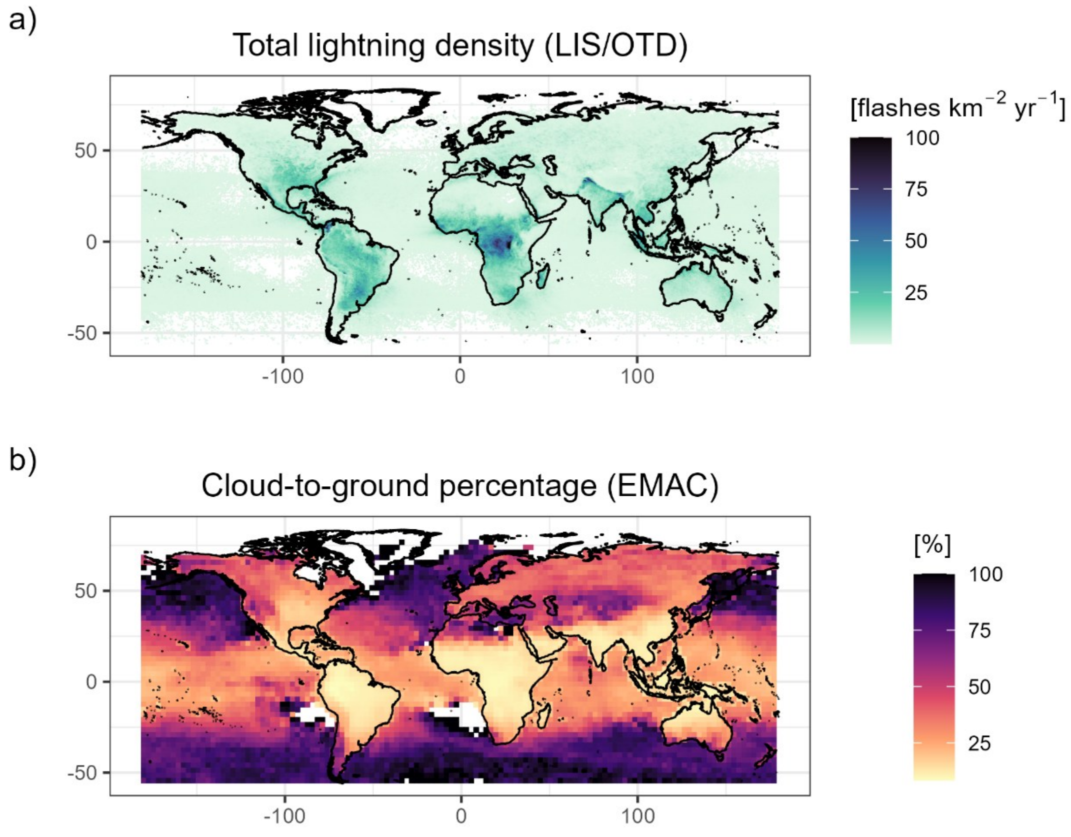

Figure S4: Total lightning density as detected by LIS/OTD (a) and cloud-to-ground percentage as simulated by the EMAC chemistry-climate model.

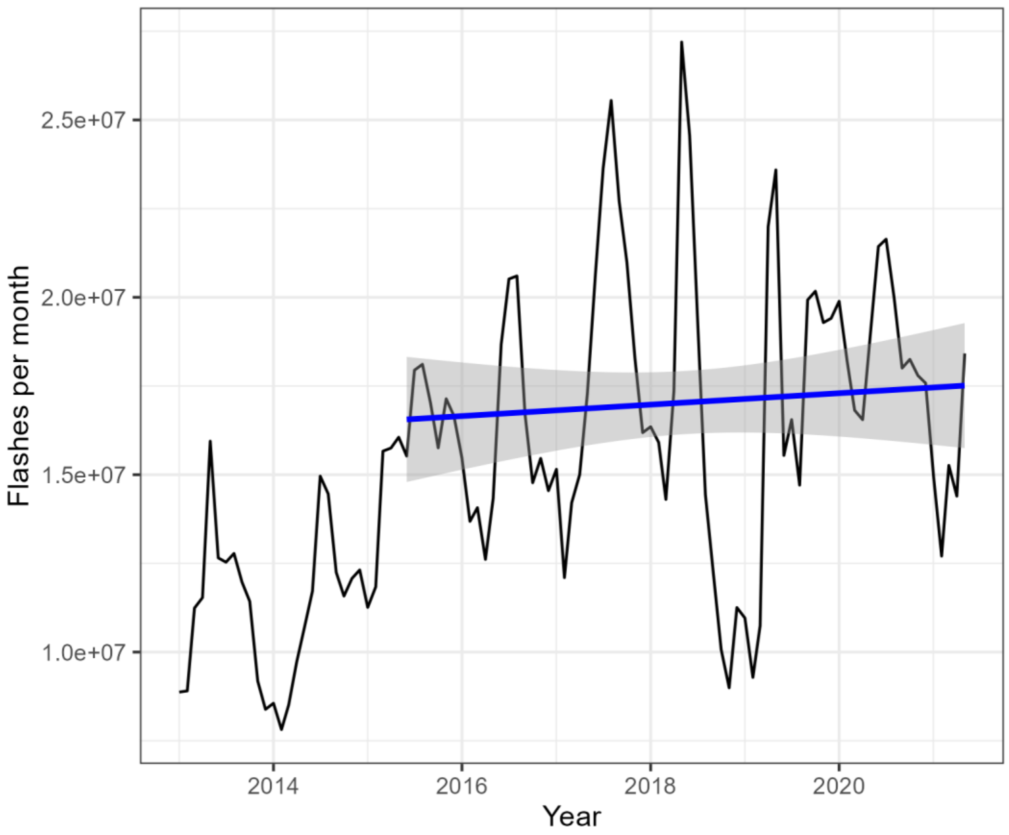

Fig. S5: Time series of ENTLN lightning frequency over land. The blue line shows the trend over the selected 06/2015-05/2021 period.

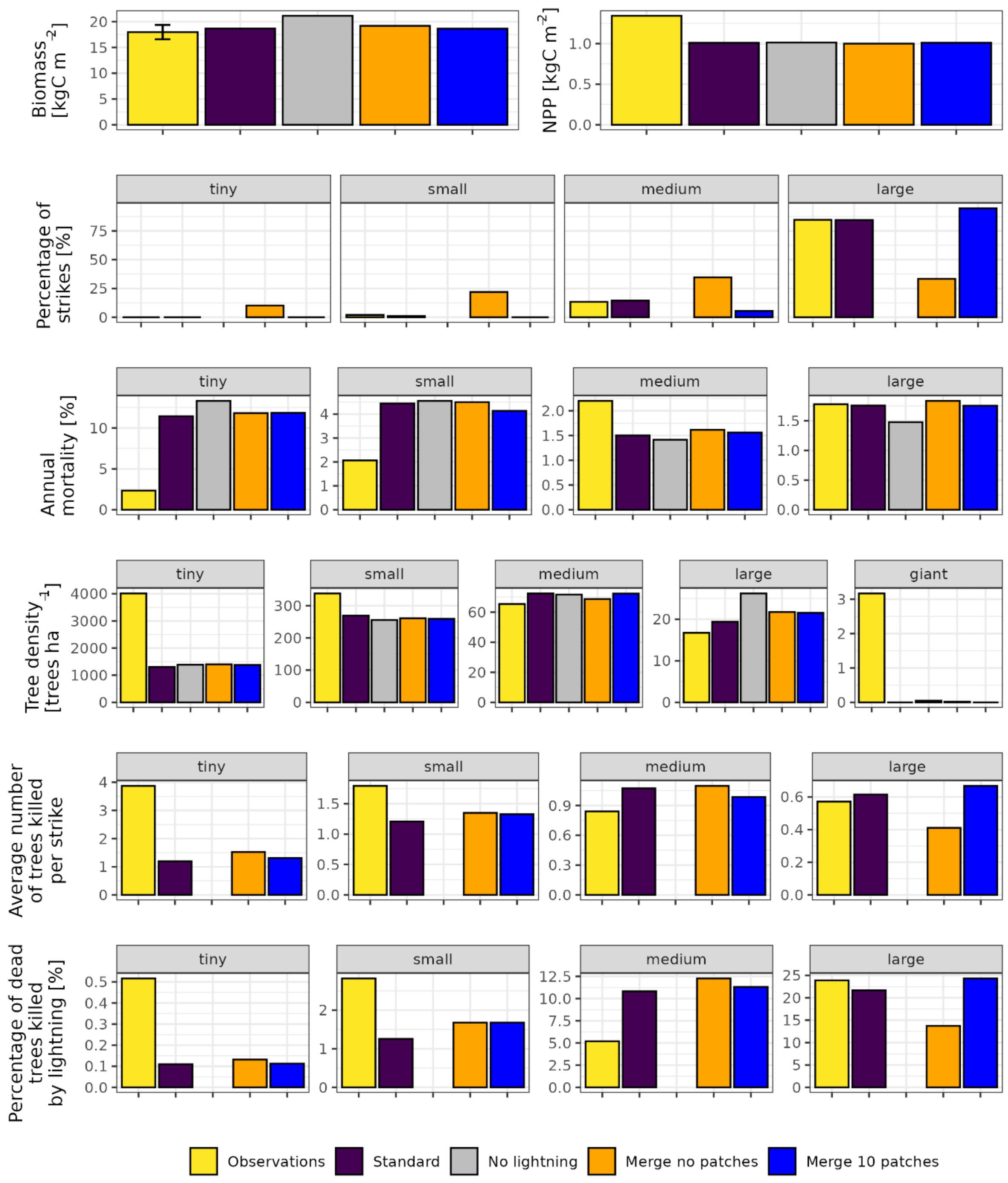

Fig. S6: Simulation results from the sensitivity analysis compared to observations. To convert observed above-ground biomass to total vegetation carbon we assumed a carbon content of 50% and an above-ground biomass fraction of 80%. For NPP, we assumed that above-ground NPP is 67% of total NPP. Note that all simulations here were done with 1000 patches so the “Standard” results differ slightly from Fig. 4.

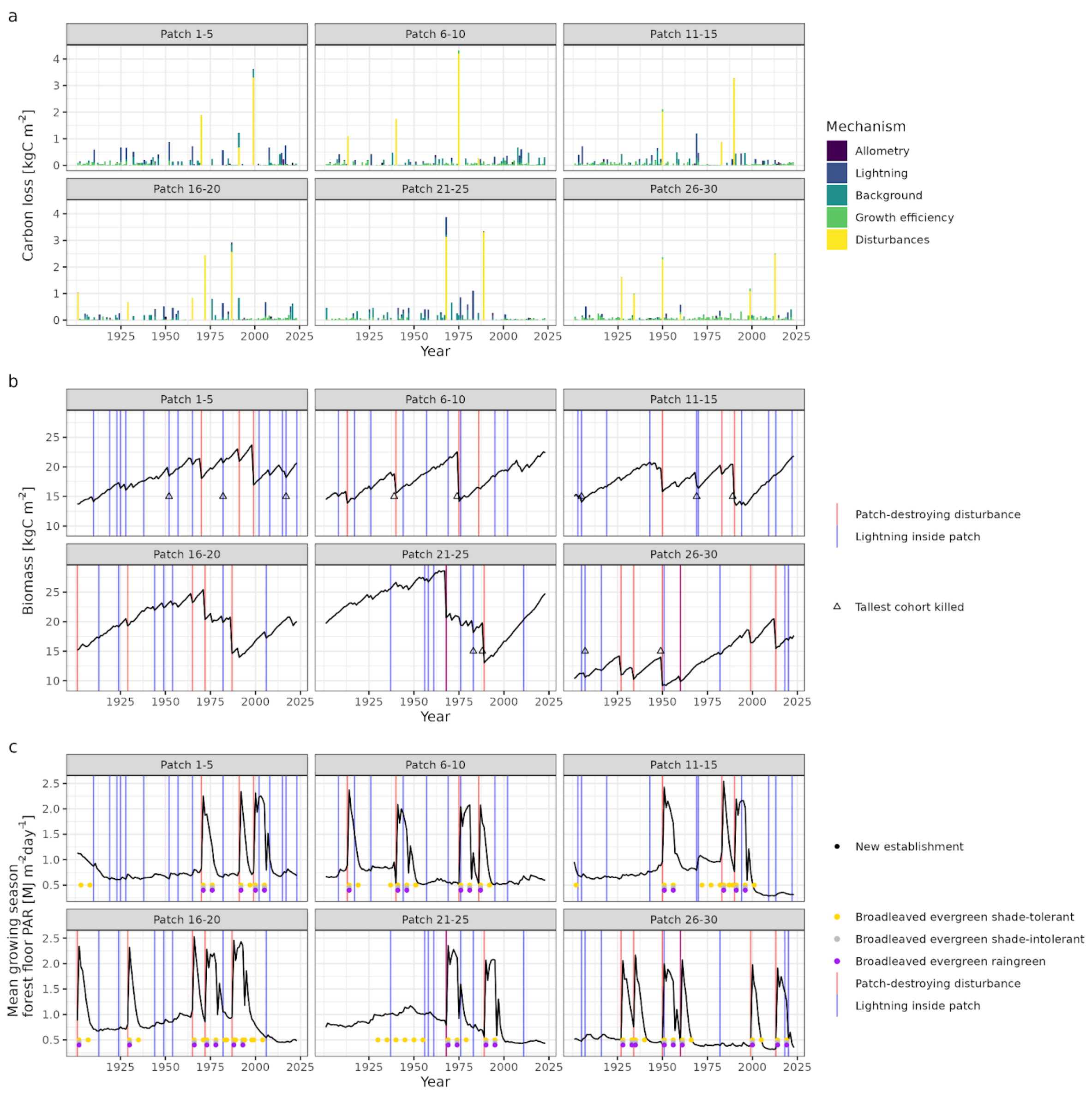

Fig. S7: Long-term impacts of lightning strikes on vegetation at patch level in Panama. The largest carbon losses are associated with occasional patch-destroying disturbances (stochastic events representing disturbances such as windthrows or insect outbreaks), while lightning mortality occurs more frequently but is less severe (a). Total biomass often decreases after a lightning strike (blue vertical lines indicate strikes inside of the patch; the less impactful strikes outside of the patch are not displayed for clarity), and, in some cases, the largest tree cohort disappears (b). However, impacts on Photosynthetically Active Radiation at the forest floor are in most cases limited and the establishment of new cohorts usually occurs only following patch-destroying disturbances (c). Note that respectively five patches are merged to align with our implementation of lightning mortality. Also note that the raingreen PFT generally dies a few years after establishment.

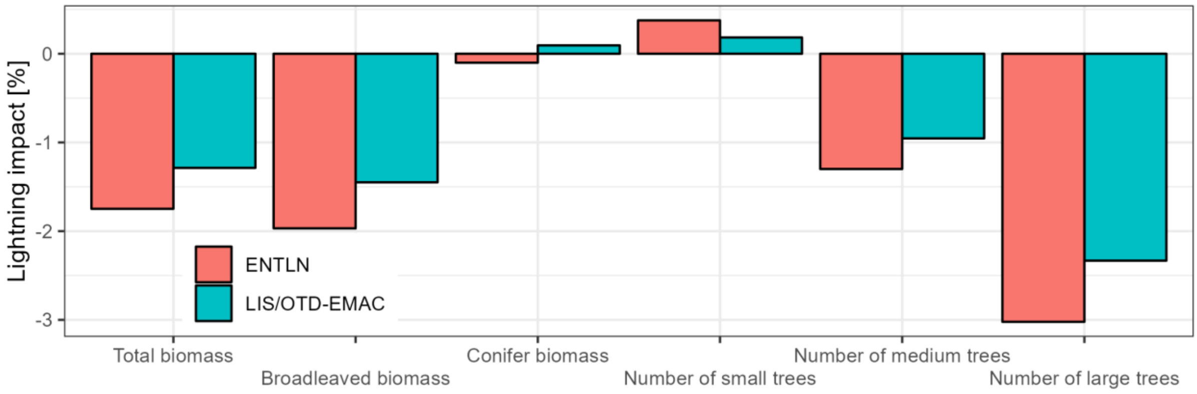

Fig. S8: Lightning impacts on global living biomass and number of trees. The impacts are computed by comparing the two simulations with lightning to the simulation without lightning.

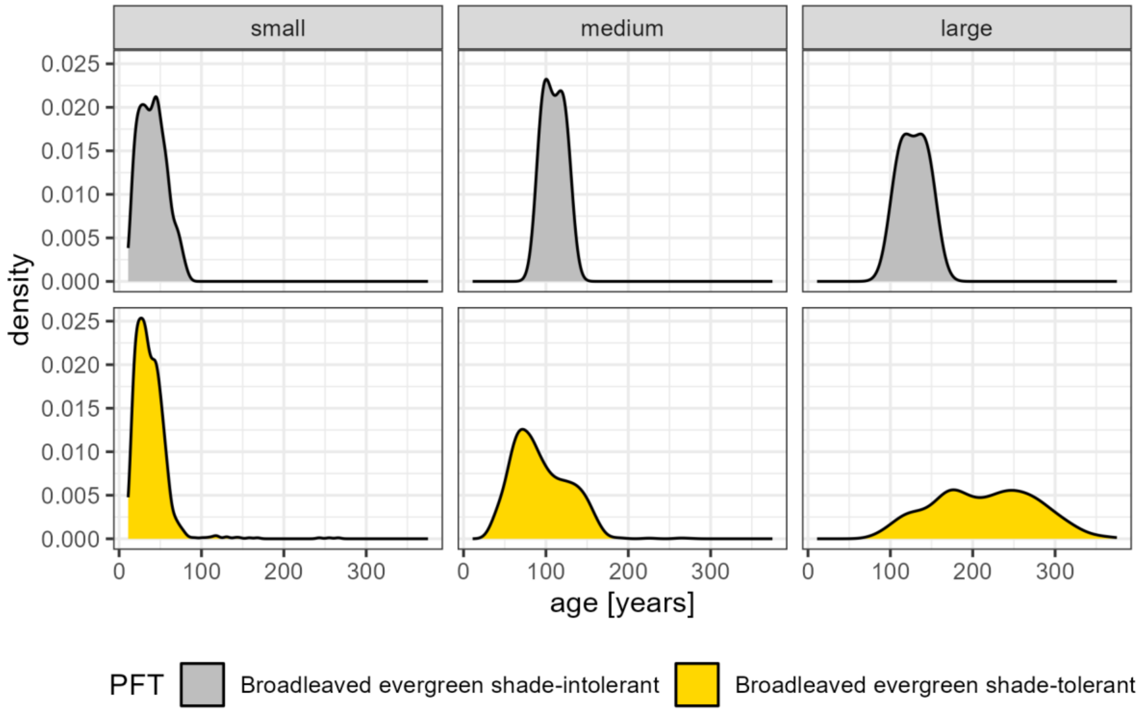

Fig. S9: Simulated tree age distribution for different size classes in Panama.

References

Conner, R. N., Rudolph, D. C., Kulhavy, D. L., & Snow, A. E. (1991). Causes of mortality of red-cockaded woodpecker cavity trees. *Journal of Wildlife Management, 55*(3), 531-537. doi:10.2307/3808986

Das, A. J., Stephenson, N. L., & Davis, K. P. (2016). Why do trees die? Characterizing the drivers of background tree mortality. *Ecology, 97*(10), 2616-2627. doi:10.1002/ecy.1497

Fontes, C. G., Chambers, J. Q., & Higuchi, N. (2018). Revealing the causes and temporal distribution of tree mortality in Central Amazonia. *Forest Ecology and Management, 424*, 177-183. doi:10.1016/j.foreco.2018.05.002

Gora, E. M., & Yanoviak, S. P. (2020). Lightning-caused disturbance in the Peruvian Amazon. *Biotropica, 52*(5), 813-817. doi:10.1111/btp.12826

Magnusson, W. E., Lima, A. P., & deLima, O. (1996). Group lightning mortality of trees in a Neotropical forest. *Journal of Tropical Ecology, 12*, 899-903. doi:10.1017/S0266467400010166

Outcalt, K. W. (2008). Lightning, fire and longleaf pine: Using natural disturbance to guide management. *Forest Ecology and Management, 255*(8-9), 3351-3359. doi:10.1016/j.foreco.2008.02.016

Palik, B. J., & Pederson, N. (1996). Overstory mortality and canopy disturbances in longleaf pine ecosystems. *Canadian Journal of Forest Research, 26*(11), 2035-2047. doi:10.1139/x26-229

Platt, W. J., Evans, G. W., & Rathbun, S. L. (1988). The Population-Dynamics of a Long-Lived Conifer (Pinus-Palustris). *American Naturalist, 131*(4), 491-525. doi:10.1086/284803

Wickham, H. (2016). *Ggplot2: Elegant graphics for data analysis*: Springer International Publishing.

Yanoviak, S. P., Gora, E. M., Fredley, J., Bitzer, P. M., Muzika, R. M., & Carson, W. P. (2015). Direct effects of lightning in temperate forests: a review and preliminary survey in a hemlock-hardwood forest of the northern United States. *Canadian Journal of Forest Research, 45*(10), 1258-1268. doi:10.1139/cjfr-2015-0081

Zoletto, B., van der Sande, M., van der Sleen, P., & Sheil, D. (2023). Lightning scars on tropical trees-Evidence and opportunities. *Ecology and Evolution, 13*(6). doi:10.1002/ece3.10210
